# Medical Advertising in Japan

**Published:** 1909-09-11

**Authors:** 


### Medical Advertising in Japan.

When the occidental world awoke five or six years ago to the fact that a new power had arisen in the far East capable of engaging successfully both by land and sea the forces of one of the largest empires of the world, not the least amazing of the facts which emerged was that even down to the army medical service the Japanese were independent of outside assistance. A good many European surgeons in search of military adventure and surgery made their way to Tokio, only to be told that their services were not required, and to wend slowly home again. We may take it that, since the military medical service was thus fully equipped with personnel, the needs of the civil population are probably also fairly well satisfied in the matter of home-bred (and mostly home-educated) practitioners. With this modernisation have entered less desirable products of western therapeutic enterprise, including the advertising quack, the patent medicine, the cure-all nostrum, and other devices by which the ignorant and the credulous have so long been bled by ingenious knaves in Europe and Great Britain. It would appear that the dimensions of these evils have grown to such an extent that the Government of the Mikado, with a praiseworthy concern for the welfare of his subjects, has taken at least the preliminary steps towards the regulation of a part of the evil. We do not gather that so far anything is being undertaken on the lines of the excellent New Zealand legislation against quack advertisements and lying nostrum vendors; but, according to an Ordinance recently published by the Home Department, very stringent rules are to be enforced with regard to the conduct of the medical profession. It is further to be observed that in respect of what shall in the future, and what shall not, be conduct befitting a Japanese doctor, the model which the regulations follow is in the main that set by the General Medical Council of Great Britain. In future no licensed medical practitioner will be permitted to advertise in Japan details of methods of medical treatment, or the history or success of such methods. Doctors and dentists connected with hospitals or engaging in general practice will not be allowed to advertise any information beyond that indicating their degrees and specialities. In this respect the Ordinance approximates perhaps more to the American idea of what is legitimate; for it is quite common to find in transatlantic journals small rectangular spaces containing the name, address, and telephone number of some practitioner, with an indication of the branch or branches of work in which he claims to be especially adept and instructed.

But after all, in regulating the extent to which qualified men may bring to public notice the fact that the State recognises their special claims to be regarded as trustworthy practitioners of medicine or surgery, the Home Department is dealing with the fringe only of a very large evil. It is something that a start should be made, but to command anything like complete success the much greater question of fraudulent cures and the immorality with which they are advertised in the lay press must be dealt with. To lay down rules for the guidance of the medical profession is much less essential than to protect the public from the unscrupulous and unqualified impostors who bolster the sales of their cure-alls by wanton lies. The Japanese Government is to be congratulated if it has decided to take steps towards the remedy of these evils; and it might well be recommended to study the penalties enacted in New Zealand not only against those who concoct quack nostrums and advertise them with false statements, but also against those who publish them without taking reasonable steps to assure themselves of the genuineness of the advertisements and the reputations of the advertisers.

